# Dimensional Roadmap for Maximizing the Piezoelectrical Response of ZnO Nanowire-Based Transducers: Impact of Growth Method

**DOI:** 10.3390/nano11040941

**Published:** 2021-04-07

**Authors:** Andrés Jenaro Lopez Garcia, Mireille Mouis, Vincent Consonni, Gustavo Ardila

**Affiliations:** 1 University Grenoble Alpes, Univ. Savoie Mont Blanc, CNRS, Grenoble INP, IMEP-LaHC, F-38000 Grenoble, France; andres-jenaro.lopez-garcia@grenoble-inp.fr (A.J.L.G.); mouis@minatec.grenoble-inp.fr (M.M.); 2 University Grenoble Alpes, CNRS, Grenoble INP, LMGP, F-38000 Grenoble, France; vincent.consonni@grenoble-inp.fr

**Keywords:** finite element method, piezoelectric sensor, mechanical energy harvesting, nanogenerator, surface Fermi level pinning, surface traps, chemical synthesis, doping level

## Abstract

ZnO nanowires are excellent candidates for energy harvesters, mechanical sensors, piezotronic and piezophototronic devices. The key parameters governing the general performance of the integrated devices include the dimensions of the ZnO nanowires used, their doping level, and surface trap density. However, although the method used to grow these nanowires has a strong impact on these parameters, its influence on the performance of the devices has been neither elucidated nor optimized yet. In this paper, we implement numerical simulations based on the finite element method combining the mechanical, piezoelectric, and semiconducting characteristic of the devices to reveal the influence of the growth method of ZnO nanowires. The electrical response of vertically integrated piezoelectric nanogenerators (VING) based on ZnO nanowire arrays operating in compression mode is investigated in detail. The properties of ZnO nanowires grown by the most widely used methods are taken into account on the basis of a thorough and comprehensive analysis of the experimental data found in the literature. Our results show that the performance of VING devices should be drastically affected by growth method. Important optimization guidelines are found. In particular, the optimal nanowire radius that would lead to best device performance is deduced for each growth method.

## 1. Introduction

With the rapid advancement in smart wearable systems and biomimetic robot technology, piezoelectric nanogenerators (PENGs) have received significant attention, for instance in the field of self-powered sensors [1] and artificial skin [2]. PENGs can be used as energy harvesting devices or mechanical sensors that convert the mechanical energy available in ambient environment (human motion, vibration, wind, etc.) into electric energy [3]. The first PENG based on ZnO nanowire (NW) arrays was built by Wang et al. [4], achieving an energy conversion efficiency between 17 and 30%. Since then, several groups have worked on PENGs based on piezoelectric nanostructures with different configurations, in particular laterally integrated nanogenerators (LING) [5,6] and vertically integrated nanogenerators (VING) [6,7,8,9,10], this last one being the most commonly used configuration due to its easy manufacturing process and its high performance [11]. The VING configuration consists of an array of vertical NWs, grown on a flexible or rigid substrate and immerged into a dielectric matrix, contacted by bottom and top electrodes. Depending on the substrate, the devices can be operated under bending [12,13,14] or compressive forces [6,7,8,9,10], the compressive mode being so far the most widely studied one. In order to quantify the output potential in compressive mode, most reports have used open-circuit conditions and evaluated the voltage generated under a mechanical load. In the case of VING devices integrated on a rigid substrate, Xu et al. [6] have used ZnO NWs with 150 nm radius and 4 μm length embedded into a polymer matrix made of poly(methylmethacrylate) (PMMA). This device has produced an output potential of about 90 mV under an applied pressure of 6.25 MPa. In Ref. [8], the authors have reported a higher generated output potential of about 290 mV under an applied pressure of about 5 kPa on a device integrating ZnO NWs featuring 100 nm radius and 3 μm length. In another example, Zhu et al. [10] have shown that the fabrication of small units of VING devices and their connection in parallel produce an output voltage of about 35 V under an applied pressure of 1 MPa. The NWs in this last device had an estimated length of 10 µm and diameters between 60 nm and 300 nm. As for VING devices integrated on a flexible substrate, Deng et al. [15] have applied a pressure of about 1 MPa on a device integrating ZnO NWs of 50 nm radius and 600 nm length over a Kapton foil, achieving an output potential of about 350 mV. Lin et al. [16] have integrated ZnO NWs of 250 nm radius and 6 μm length on a polydimethylsiloxane (PDMS) substrate. Under a compressive strain of about 0.12%, they have produced an output voltage of about 8 V. It is important to mention that all the above-mentioned experiments used chemical bath deposition (CBD) for the growth of ZnO NWs, as an easy-to-implement and low temperature process compatible with industrial applications.

In the literature, there has been very few articles reporting the effect of ZnO NW dimensions on the performance of VING or related devices, despite their critical role for optimization. Rivera et al. [17] have investigated the generation of electrical energy from ZnO NW arrays as a function of their length using a charge amplifier. They have reported that the longest NWs, with 4.7 µm in length and 130 nm in diameter, produce the highest energy of about 35 nJ. In contrast, the shortest ones, with 1.3 µm in length and 100 nm in diameter, generated an energy of only 10 nJ. Kammel et al. [18] have investigated NW length influence on the output potential of VING devices. Their device consisted of a double-sided ZnO NW array covered by PDMS. They reported that the longest NWs, with an average of 2.4 µm in length and 66 nm in diameter, produce the highest values of output potential (about 4.48 V) when a pressure was exerted on the device. In contrast, the shortest NWs, with an average of 1.0 µm in length and 317 nm in diameter, generated an output potential of only 1.84 V. These two experimental reports used the CBD technique for ZnO NW growth. Riaz et al. [19] have explored voltage generation from ZnO NW arrays by scanning a conductive Atomic Force Microscopy (AFM) (Pt/Si) tip over samples that had been grown by two different deposition techniques. They have shown that the longest NWs (3–5 µm long and 100–200 nm wide), grown by carbothermal reduction using a vapor-liquid-solid (VLS) mechanism, generate an output voltage of about 30–35 mV on average while the shortest ones (1–2 µm long and 200 nm wide), grown by CBD, generate an output voltage of about 5 mV.

Basically, it is not straighforward to predict the output potential generated from NW-based piezoelectric transducers nor their general performance, because several key parameters, such as geometrical dimensions (i.e., radius and length), doping level (Nd), and surface trap density (Nit) [20] all play a significant role, and their relative effect on general performance may not be readily decoupled. One reason for that is that these key parameters strongly depend on the growth method used to form ZnO NWs. In the field of compound semiconductors, ZnO is a unique material since it can be formed in the shape of NWs by a very large number of growth methods using a self-assembled/self-induced approach (i.e., with no catalyst) [21]. It has been reported in the last two decades that ZnO NWs can be grown by physical vapor deposition techniques [22,23], chemical vapor deposition techniques [24,25], and wet chemistry [26,27]. On the one hand, this wide variety of growth methods offers a great opportunity to form ZnO NWs with controlled morphologies, tailored dimensions, and dedicated properties. On the other hand, the different media and chemicals used in these growth methods result in the formation of ZnO NWs exhibiting a broad range of doping level and surface trap density. Overall, it is well-known that ZnO NWs exhibit a high electrical conductivity and thus a high doping level, regardless of the growth method involved [28]. However, charge carrier density values spread over several decades from 10^17^ cm^−3^ to 10^20^ cm^−3^ [28,29,30,31,32,33,34,35,36,37,38,39,40,41,42,43,44,45,46,47,48,49,50]. The main reason is related to a large incorporation of residual impurities (i.e., Al, Ga, In, etc.) which act as shallow donors in the vapour phase deposition technique [31], as well as to the specific role of hydrogen which can form a wide variety of defects acting as shallow donors in the wet chemistry techniques [37]. Although ZnO NWs exhibit a top polar c-face and six non-polar m-plane sidewalls regardless of the growth methods used, the main characteristics of these surfaces (e.g., surface roughness) are not equivalent. Surface trap density is also, to a significant extent, affected by the growth method used [28,33,49,51,52]. It is thus very important to take the growth method into consideration in the design and optimisation of piezoelectric mechanical transducers in view of its effect on doping level and surface trap density. However, this line of research has not been explored so far.

From a theoretical point of view, the geometrical parameters of ZnO NWs have been shown to affect their overall piezoelectric performance [53,54]. These studies reveal that thinner and longer NWs would increase the generated piezoelectric potential when sollicited under compressive forces, but they do not consider their semiconducting properties, which represent a strong limitation. The influence of doping level and free carrier screening on the piezoelectric response of semiconducting (mostly ZnO) NWs has been studied theoretically by means of numerical simulations [55,56,57]. The presence of free charge carriers in the core of NWs basically screens the piezoelectric potential induced by the strain generated under mechanical solicitations. In these simulations, single ZnO NWs have been simulated under a compressive force. Compression creates a depletion region at the top of the NW. The piezoelectric potential can be generated only in this depleted region, making the performance practically independent of NW length [56]. These reports have also shown that an increase in doping level reduces the piezoelectric response. In Ref. [57], the authors added an external surface charge density at the top of the NW, extending the depletion region from the top and thus increasing the piezoelectric response of the NW but keeping it independent of length for practical length values. Experimental reports have confirmed the role of doping level on the VING device performances [58]. However, they have also demonstrated a dependence of the performance on NW length [17,18,19] which could not be explained by these theoretical models. It is then very important to correlate theoretical models and experiment data to elucidate the key parameters affecting the device performance. Understanding the effect of those parameters will allow the development of guidelines for the design of new devices along with their optimization.

It has been shown recently by numerical simulation that Fermi level pinning, resulting from the presence of surface traps at the interface between ZnO and the matrix material could explain the experimentally observed length dependence [20]. The presence of surface and interface traps has been widely acknowledged in III–V and II–VI semiconductors [59,60,61,62]. Their impact on device operation has been explained as follows [20]. In the absence of surface traps, or for low trap densities, there is no surface charge, and thus no band bending at the surface of ZnO. In this case, the application of a pressure to the VING generates a polarization field that depletes only the top of the NWs, while the core of the NW remains neutral, with free carriers screening the polarization field. This is known as the free surface Fermi level assumption, which is the usual assumption in most numerical simulations [56,57]. However, in the presence of a large density of surface traps along the NW (m-plane) sidewalls, which is expected in ZnO NWs [63], thermodynamic equilibrium results in surface band bending, with a balance between surface charges and depletion charges (Figure S1 in the Supplementary Materials). If the surface trap density is large enough, surface Fermi level can be considered as pinned close to mid-gap. Therefore, depending on NW diameter, doping level and surface trap density, it may become possible to deplete the NW until its core so that its whole volume contributes to the generated piezoelectric potential. It has been shown that provided surface traps can be considered as slow enough, they contribute to performance improvement by suppressing free carrier screening [20].

In the present work, we theoretically explore the role of free charge carriers, surface traps, and NW dimensions on the output potential of piezoelectric nanocomposites based on ZnO NWs with account for the latitude of variation offered by growth methods. The properties of NWs grown by the most widely used methods including thermal evaporation (TE), chemical vapor deposition (CVD), metal-organic CVD (MOCVD), and CBD with either O- or Zn-polarity are obtained from a thorough and comprehensive analysis of the experimental reports found in the literature. The theoretical investigation is carried out for a typical VING configuration integrating vertical ZnO NWs embedded into a polymer matrix material (PMMA here). The device is considered as operated in compression. This configuration is chosen as it corresponds to most of the experimental reports, but the conclusions drawn are more general. The theoretical study is performed with a finite element method (FEM) approach by solving the full set of coupled equations, describing the mechanical, piezoelectric and semiconducting properties of the structure. Important optimization guidelines are found, concerning in particular the optimal NW radius needed for each growth method in order to obtain the best device performance and specifically to maximize the output piezoresponse. The effect of the variation of the length is also analyzed, in particular for the NWs with O-polarity grown by CBD, which are offering a great potential in the field of piezoelectric devices.

## 2. Simulation Framework

In this work, we simulated the full set of differential equations that couple mechanical, piezoelectric, and semiconducting properties using the FEM approach. To this end, we used the FlexPDE^®^ environment, which provides fully flexible description of geometry, differential equations to be solved, and boundary conditions.

### 2.1. Device under Study and Simulated Structure

**Figure 1:**
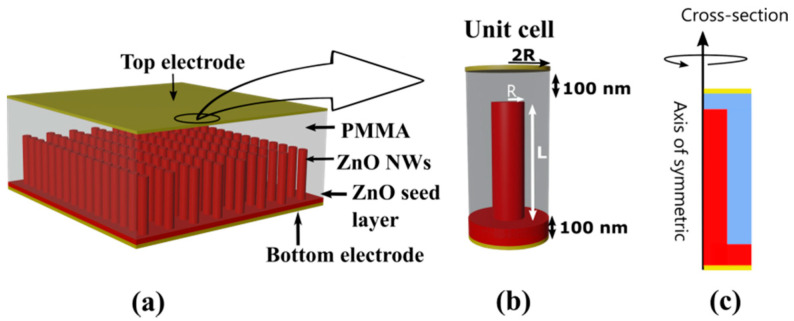
(**a**) Structure of the VING device; (**b**) unit cell of the VING and (**c**) cross section of the VING unit cell around the axis of symmetry.

The classical VING structure is depicted in Figure 1a. It is composed of an NW-based active layer, a polymer layer, and two metallic electrodes placed at the top and bottom of the structure. The active layer is typically made of a thin ZnO seed layer (several 10 nm thick) covered with a self-organized array of vertical ZnO NWs (several µm long). PMMA is typically used as matrix material to encapsulate the NWs and make the structure mechanically robust, as well as to isolate the NWs and avoid current leakages. The device works in capacitive mode. When an external mechanical load is applied on top of the device (a vertical compression in this case), strain is transferred to the active material. The input strain produces dipoles inside ZnO by the direct piezoelectric effect and a polarization field is then created charging the external electrodes.

The simulation of a VING integrating billions of NWs, arranged in a pseudo-periodic disordered array, would require an extremely high computational cost. We simplified the approach by adopting a standard strategy, already used to simulate ZnO composites under compression and bending conditions [20,54], which consists in restricting simulation space to a unit cell made up of a single ZnO NW surrounded by an insulating matrix (PMMA in this case) over a ZnO seed layer. The unit cell is sufficient to determine the generated piezoelectric potential of a whole device sollicited under compression with a high precision if the appropriate boundary conditions are applied in the model [20,54]. Furthermore, a 2D axisymmetric cylindrical model was used for the unit cell as shown in Figure 1b. The 3D cylindrical geometry which derives from the 2D axisymmetric one by rotation around the NW axis, as shown in Figure 1c, was considered as a fair enough approximation. All equations and parameters were transformed from Cartesian to cylindrical coordinates (vertical and radial coordinates, z and r, respectively).

In this work, we fixed the thickness of the ZnO seed layer to 100 nm and that of the thick insulating cap layer (PMMA) to 100 nm as well. The radius (R) of the ZnO NW was varied consistently with what is achievable with the different growth methods. The width of the unit cell was defined as two times the NW diameter (Figure 1b) based on previous work [54]. The effect of NW length (L) on device performance was also studied. The parameters related to semiconducting properties, namely doping level (N_d_) and surface trap density at the interface between ZnO and PMMA (N_it_), were also varied as a function of growth method.

### 2.2. System of Equations

In order to calculate the output electric potential generated by the VING device under compression, we solved the coupled system of equations for a system based on n-type semiconducting ZnO NW with piezoelectric properties, as in [20]:

(1) $$\nabla \left(\left[c\right]\left[\varepsilon \right]\right)+\nabla \left({\left[e\right]}^{T}\vec{\nabla }V\right)=0,$$

(2) $$\nabla \left(\left[\kappa \right]\vec{\nabla }V\right)-\nabla \left(\left[e\right]\left[\varepsilon \right]\right)\;=\rho ,$$

where *V* is the electric potential, [c] is the elasticity matrix, [e] is the piezoelectric coefficient matrix, [κ] is the dielectric constant matrix, and [ε] is the strain matrix. The second terms on the left-hand side of Equations (1) and (2) represent the piezoelectric coupling terms, and ρ is the local charge density given by:

(3) $$\rho =\{\begin{array}{c} q\left(n-N_{d}\right)\;\mathrm{in}\;\mathrm{semiconducting}\;\mathrm{regions}\\ 0\;\;\;\mathrm{in}\;\mathrm{insulating}\;\mathrm{regions} \end{array},$$

where q is the electron charge and N_d_ is the concentration of ionized donor atoms. In previous studies [20], the free carrier concentration n was computed using Boltzmann statistics. However, depending on growth method, doping level can reach quite large values with respect to degeneracy level, which is around 10^18^ cm^−3^ in ZnO. Here, *n* was thus computed by considering Fermi-Dirac statistics:

(4) $$n=N_{d}F_{\frac{1}{2}}\left(\frac{qV}{k_{B}T}\right),$$

where F_1/2_ (x) is the Fermi-Dirac integral function of order ½, kB is the Boltzmann constant, q is the electric charge of one electron, and T is the temperature, considered here equal to 300 K.

The variation of the output potential resulting from the variation of external pressure on the device was obtained by solving Equations (1) and (2) for two cases: first, for the initial state (i.e., without compression) and then for the final state (i.e., under vertical compression). The output potential or “piezoresponse” in the results section was then calculated as the potential difference defined as *V*_Final state_ − *V*_Initial state_.

### 2.3. Boundary Conditions

**Figure 2:**
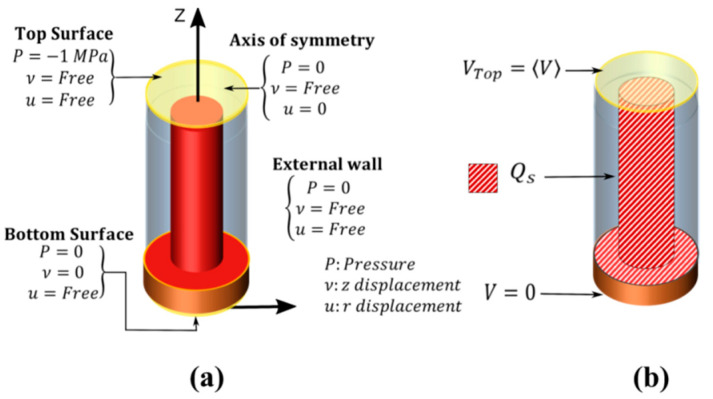
(**a**) Mechanical and (**b**) electrical boundary conditions of the VING unit cell.

The mechanical boundary conditions consisted in (i) free vertical displacement and forbidden lateral displacement on the axis of symmetry, (ii) a vertical pressure of -1MPa along the NW axis (z-axis) with free vertical and lateral displacement on top surface, (iii) free lateral displacement with forbidden vertical displacement at the bottom, (iv) free lateral and vertical displacement on the outer lateral side (Figure 2a).

In terms of electrical boundary conditions (Figure 2b), the bottom electrode was grounded, while Neumann conditions were applied to other boundaries. The top electrode potential *V*_Top_ was obtained by averaging *V* on the top surface. At the interface of ZnO and PMMA (i.e., the diagonal line pattern on the ZnO surface from Figure 2b), we introduced a surface charge Q_s_ under the assumption of a uniform trap density (N_it_) at thermal equilibrium. In this paper, the potential used to calculate Q_s_ was taken from the initial state, which simulates ideally slow traps, with a charge that remains frozen during the transition from initial to final state. Q_s_ was expressed as a function of the local potential *V*_init_ as:

(5) $$Q_{s}=\;-q^{2}{\;N}_{\mathrm{it}}\;\left(V_{\mathrm{init}}-\varphi_{\mathrm{Fi}}\right),$$

where φ_Fi_ is the difference between Fermi level and intrinsic level.

## 3. Simulation Results and Discussions

### 3.1. Input Experimental Data for the Simulation

**Figure 3:**
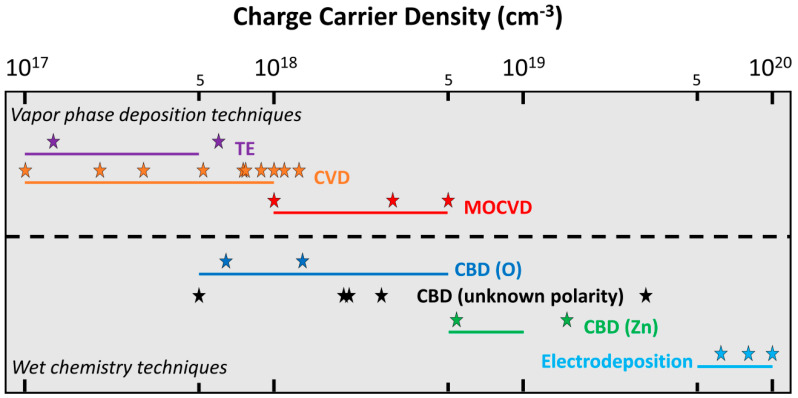
Schematic diagram summarizing experimental data for charge carrier density in ZnO NWs grown by TE [29,40], CVD [28,44,45,46,47,48,49,50], MOCVD [31,32,33], CBD (O) [36], CBD (unknown polarity) [34,37,43], CBD (Zn) [36], and electrodeposition [38,39,41]. A logarithmic scale is used for doping level. The coloured solid stars represent the experimental data point reported in the literature for each growth method, as an average value [28,29,33,34,38,39,40,41,44,49,50] or an interval of minimum and maximum values [31,32,36,37,43,45,46,47,48]. The coloured solid lines represent the deduced range of charge carrier density used in the numerical simulation for each growth method.

A set of numerical simulations of VING devices integrating ZnO NWs was performed considering the characteristics of typical NWs grown by each method using standard conditions, namely without any intentional doping and post-deposition treatment: a typical range of radius (R), a typical length value, a typical range of doping level (N_d_), and a typical surface trap density (N_it_) value were selected in that purpose. As regards the dimensions of ZnO NWs, their typical radius was varied over a similar range of 4 to 150 nm while their length was firstly kept fixed to 5 µm in all devices. These dimensional properties of ZnO NWs are very typical and similar for each growth method. In contrast, the range of doping level in ZnO NWs strongly depends on the growth method used, as represented in Figure 3. From the large number of experimental data reported in the literature using field-effect transistor (FET) measurements [30,35,40,44,45,46,47], I–V measurements on four-terminal contacted ZnO NWs [28,29,31,36,37,50], terahertz spectroscopy [34], conductive AFM (i.e., SSRM and SCM measurements) [32,43], and electrochemical impedance spectroscopy [39,41], a range of charge carrier density values was inferred for each growth method when ZnO NWs are grown using standard conditions (i.e., typical chemical precursors, typical growth temperature and pressure). Overall, the charge carrier density of ZnO NWs typically lies in the range of 10^17^ to 10^20^ cm^−^^3^. The vapour phase deposition techniques including TE, CVD, and MOCVD methods result in the formation of ZnO NWs with a lower mean charge carrier density ranging from 10^17^ to 5 × 10^18^ cm^−3^ at maximum [28,29,30,31,32,33,40,44,45,46,47,48,49,50]. In present deposition techniques, the incorporation of residual impurities (i.e., Al, Ga, In) acting as shallow donors is mainly responsible for this range of charge carrier density values [31]. Residual impurities usually occur as contaminants in the materials sources (i.e., TE) or in the growth chamber (i.e., CVD, MOCVD). The high growth temperature used in MOCVD is also favourable to the diffusion of residual impurities from the substrate, like Al from sapphire, into ZnO NWs. In contrast, wet chemistry deposition techniques, including CBD and electrodeposition, lead to the formation of ZnO NWs with a higher mean charge carrier density, ranging from 5 × 10^17^ cm^−3^ at minimum to 10^20^ cm^−3^ [34,35,36,37,38,39,41,42,43]. In the electrodeposition process, the use of zinc choride as the typical chemical precursor to enhance the morphology of ZnO NWs is favourable to the massive incorporation of chlorine acting as a shallow donor [42]. In the CBD process, the massive incorporation of hydrogen-related defects acting as shallow donors is mainly responsible for this range of charge carrier density [36]. The growth medium in water is full of hydrogen and the crystallization process resulting in the elongation of ZnO NWs through the development of their c-plane top facet basically involves a dehydration process [64]. A large number of hydrogen-related defects (e.g., interstitial hydrogen, substitutional hydrogen on the oxygen lattice site, zinc vacancy–hydrogen complexes) acting as shallow donors are thus formed systematically [37].

The CBD technique further offers a unique opportunity to form either O– or Zn-polar ZnO NWs [65] with a significant difference in their electrical conductivity and both of them were considered in the present theoretical investigation [36]. This is in strong contrast with physical vapour deposition techniques, for which ZnO NWs are systematically of Zn polarity when the self-assembled/self-induced approach is employed [66]. In contrast to the large number of experimental data reporting the doping level of ZnO NWs, the surface trap density and its dependence on each growth method have been much less investigated experimentally. Only a couple of investigations have been achieved, mainly by steady-state and time-resolved optical spectroscopy through the determination of the surface recombination velocity [52]. However, a consensus in the literature seems to emerge that the surface trap density of ZnO NWs grown by physical vapour deposition techniques is about 2–3 × 10^12^ cm^−2^ (i.e., about 10^12^ eV^–1^ cm^−2^) [28,33,49,51]. In contrast, the surface trap density of ZnO NWs grown by wet chemistry is about one decade larger (1–4 × 10^13^ cm^−2^ i.e., about 10^13^ eV^–1^ cm^−2^) [52]. The higher value when using wet chemistry is expected because their surfaces are typically rougher and thus present a larger density of defects.

### 3.2. Piezoelectric Performance as a Function of the Nanowire Growth Method

The piezoresponse was calculated for each device for a fixed NW length of 5 µm, variable NW radius and taking into account typical values of N_d_ and N_it_ corresponding to each NW growth method, as described in the previous section. A numerical simulation was also made to evaluate the effect of varying the length (L) for one particular growth method that is of high interest, namely the CBD growth of O-polar ZnO NWs, and with a typical radius set to 50 nm.

#### 3.2.1. TE Method

**Figure 4:**
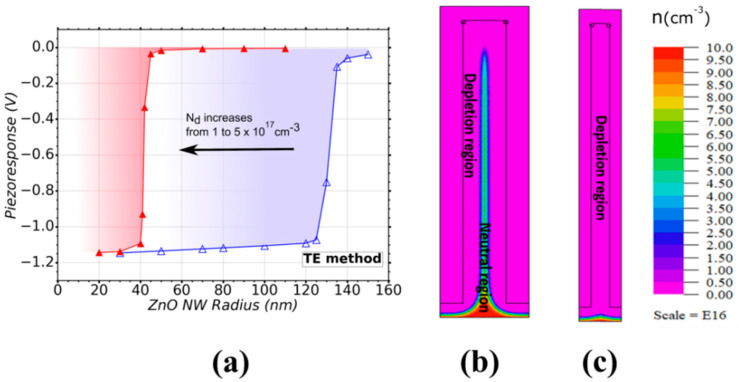
(**a**) Variation of the piezoresponse of a VING as a function of the ZnO NW radius for a range of doping level (N_d_) from 1 (△) to 5 (▲) × 10^17^ cm^−3^ typical in the TE method. The shadowed zones represent the regions of interest for a given doping level. Free carrier distribution of a NW with N_d_ = 1 × 10^17^ cm^−3^ and a radius of (**b**) 140 nm and (**c**) 80 nm. A trap density N_it_ = 1 × 10^12^ eV^–1^ cm^−2^ was considered in the simulation.

Figure 4a shows the calculated piezoresponse of a VING transducer for different radii of ZnO NWs ranging from 20 nm up to 150 nm. A negative piezo response is obtained as expected [54] because the *c*-axis in the NW is oriented along the [0001] direction (i.e., Zn polarity) [67]. This is the case in the vast majority of the ZnO NWs grown by the numerous physical and chemical techniques we investigate here. With this growth method, Nd was evalutated in the range from 1 (blue curve) to a maximum value of 5 (red curve) × 10^17^ cm^−3^ and a value of N_it_ = 1 × 10^12^ eV^–1^ cm^−2^ was taken. These parameters correspond to ZnO NWs grown by the TE method as presented in Figure 3. Figure 4a shows a strong radius dependence of the piezoresponse, further influenced by the doping level. At the minimum value of N_d_, a step in the piezoresponse was observed, with a strong increase (by about 13 times) in absolute value, as NW radius was reduced from 140 nm to 120 nm. A further reduction of the radius had little effect on the piezoresponse. A similar dependence was also found for the maximum value of N_d_, but the step in piezoresponse was observed at lower radius (i.e., for a reduction from 50 nm to 40 nm). The region between the two curves in Figure 4a thus represents the range of optimization of the VING devices. VING devices integrating NWs with a radius larger than 120 nm for low doping levels (blue curve), and larger than 40 nm for high doping levels (red curve), result in poor piezoresponse. This is due to the screening effect originating from free carriers in the ZnO NW [20,54,56,68,69,70]. An example of this effect is shown in Figure 4b. It depicts a qualitative map of the free carrier distribution in a VING device under compression. The VING transducer integrates NWs with a radius of 140 nm and a low doping level (1 × 10^17^ cm^−3^). A depletion region is created from the PMMA/ZnO interface and a neutral core starts from the bottom and extends towards the top of the NW. In this neutral region, the free carriers screen the piezoelectric response and the contribution of polarization electric charges is largely reduced, thus the overall voltage is reduced as well. Figure 4c shows the effect of the reduction of the NW radius down to 80 nm at the same doping level. In this case, the depletion region is large enough to fully deplete the NWs from its sides and from its top, drastically increasing the performance of the device. We can thus identify a critical value for the ZnO NW radius, below which the performance of VING transducers can be largely improved, for given doping level and trap density. This particular NW radius will be called “NW critical radius” all along the article and summarised for every growth method in Table 1. The critical radius can be evaluated analytically from charge neutrality between surface traps and surface depletion under the additional condition that depletion region reaches the center of the NW by solving Poisson equation in cylindrical coordinates (Figure S1 and Equation S5 in the Supplementary Materials).

#### 3.2.2. CVD Method

**Figure 5:**
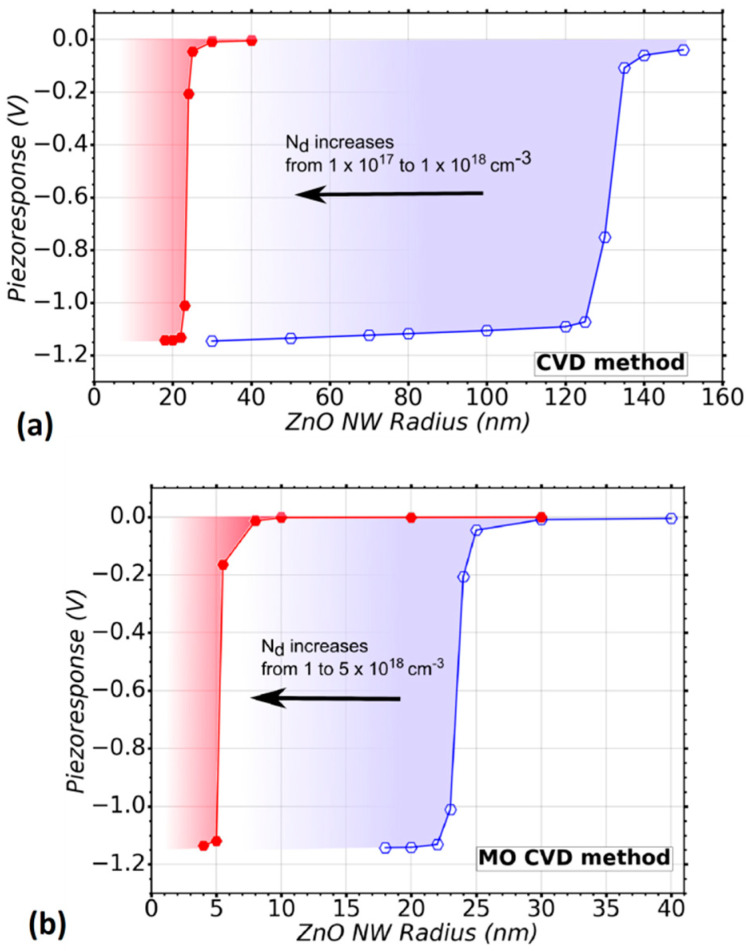
Variation of the piezoresponse of a VING as a function of ZnO NW radius taking into account two vapor deposition techniques for the growth: CVD and MOCVD. (**a**) A range of doping levels (N_d_) from 1 × 10^17^ (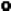) to 1 (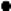) × 10^18^ cm^−3^ is considered, which is typical in the CVD method, (**b**) a range of doping level (N_d_) from 1 (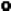) to 5 (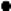) × 10^18^ cm^−3^
is considered, which is typical in MOCVD method. A trap density N_it_ = 1 × 10^12^ eV^−1^ cm^−2^ was considered in the simulations.

To further explore the influence of the growth method of the NWs on the VING performance, the piezoresponse was also calculated using the characteristics reported for ZnO NWs grown by the CVD method. These NWs present lower values of radius and higher doping level as compared to the NWs grown by TE although they present equivalent values of N_it_. The doping level lies in the range from 1 × 10^17^ up to 1 × 10^18^ cm^−3^, corresponding to the blue and red curves in Figure 5a, respectively. Figure 5a shows the weak piezoresponse obtained with NWs exhibiting radii larger than about 140 nm for low doping levels (blue curve) and 25 nm for high doping levels (red curve). A higher performance was obtained for NW radii below 120 and 20 nm for low and high doping levels, respectively.

#### 3.2.3. MOCVD method

Figure 5b shows the piezoresponse of a VING device integrating ZnO NWs with the characteristics reported for the MOCVD method. In this case, N_d_ goes from 1 (blue curve) to 5 (red curve) × 10^18^ cm^−3^. This method shares the same Nit as TE and CVD methods as a first approximation. According to the results, the piezoresponse is improved when the ZnO NWs radius is smaller than 22 nm for minimum N_d_ (blue curve) and smaller than 5 nm for maximum N_d_ (red curve). A poor performance is expected for NWs with radius larger than about 30 and 7 nm for low and high doping levels, respectively. It should be noted that the simulation of a device integrating NWs with 5 nm radius could reach the limit where continuum medium equations do not apply anymore. In this sense, it should be considered as a rough approximation. Indeed, according to studies based on first-principles calculations, ZnO nanoscale materials with radius lower than 3 nm could present significantly higher piezoelectric coefficients compared to bulk material, namely there would be a radius-dependent size effect with its piezoelectric properties [71]. However, the formation of ZnO NWs with a radius smaller than 5 nm has not been experimentally shown yet and there has been thus no experimental evidence yet of the improvement of the piezoelectric coefficients in that range of radii. A comparison of the performance obtained when using these last three growth methods involving vapour phase deposition techniques (TE, CVD, MOCVD) show that smaller radii are gradually required to obtain the optimal performances: 120 nm, 120 nm, and 22 nm respectively, for low doping levels, as well as 40 nm, 20 nm, and 5 nm, respectively, for high doping levels. This can be explained as the doping level of NWs grown by MOCVD is higher than with the other growth methods, and the doping level of the NWs grown by TE is the lowest one, while keeping the same surface trap density. Because of the higher doping levels, NWs with smaller radii are required in order to obtain the full depletion of free charge carriers and thus to reduce the screening effect.

#### 3.2.4. CBD Method

**Figure 6:**
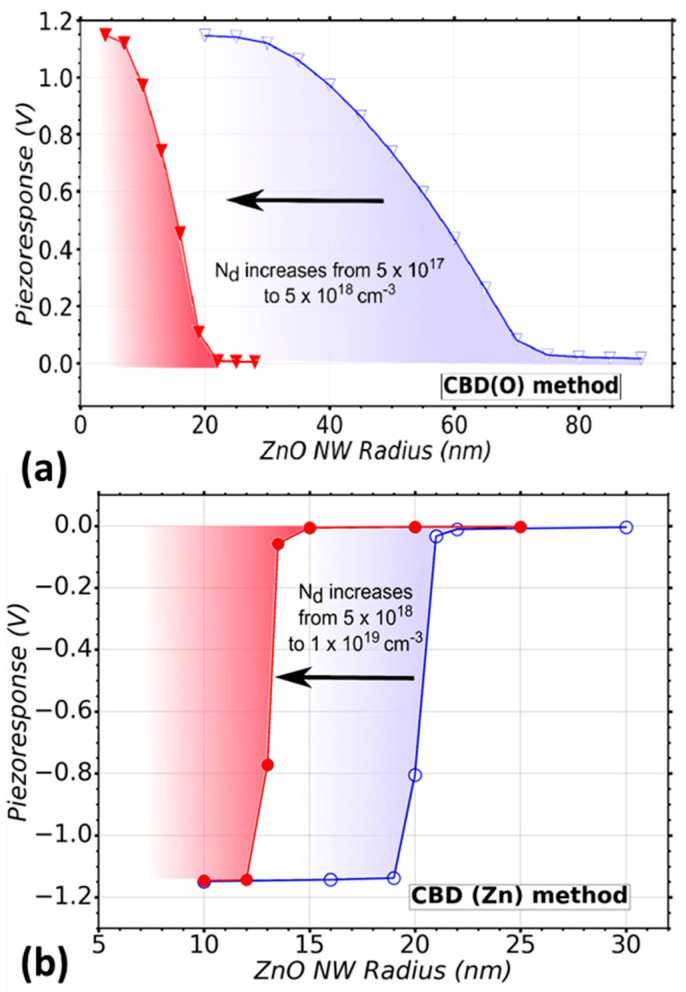
Variation of the piezoresponse of a VING as a function of ZnO NW radius taking into account the CBD deposition technique. (**a**) A range of doping level (N_d_) from 5 × 10^17^ (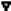) to 5 (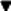) × 10^18^ cm^−3^ is considered, which is typical in CBD (O), (**b**) a range of doping level N_d_ from 5 × 10^18^ (○) to 1 (●) × 10^19^ cm^−3^ is considered, which is typical in CBD (Zn) method. A trap density N_it_ = 1 × 10^13^ eV^−1^ cm^−2^ was considered in the simulations.

The CBD technique was also considered as an important growth method for flexible devices in our theoretical study. The O- and Zn-polar NWs grown by the CBD method present a higher surface trap density compared to the previous methods (e.g., N_it_ is equal to 1 × 10^13^ eV^–1^ cm^−2^. Figure 6a shows the radius dependence of the VING performance for the CBD (O) method, namely for O-polar ZnO NWs. A positive piezo response is obtained in this case because the *c*-axis in the NW is oriented along the [000–1] direction (i.e., O polarity). This also changes slightly the interaction between the piezoelectric and semiconducting effects, producing a less sharp transition from the fully screened piezo response state to the fully depleted NW state where a maximum performance is obtained. The doping level range goes from N_d_ = 5 × 10^17^ cm^−3^ (blue curve) to N_d_ = 5 × 10^18^ cm^−3^ (red curve). Owing to the higher surface trap density of the CBD method, a better performance can be obtained for larger radius compared to that of the MOCVD method. For instance, half the optimal piezo response (~0.6V in absolute value) can be obtained for a NW grown by the CBD (O) method with a radius of ~55 nm. In contrast, a radius of ~23 nm is needed for a NW grown by MOCVD, which is larger by a factor of more than 2. Interestingly the critical radii are very similar for both methods. The best performance can be obtained for NWs with radius below 20 nm and 5 nm for low and high doping levels, respectively. For NW radius larger than 70 nm (low doping level) and 22 nm (high doping level), we expect that a poor performance is obtained.

Finally, the performance of VING devices integrating Zn-polar NWs grown by the CBD method presents a very low optimization window as compared to O-polar NWs grown by the same CBD technique (Figure 6b). The optimal performance can be obtained using NWs with radius below about 18 nm and 12 nm for low and high doping levels, respectively. This window of optimization of about 6 nm is around 9 times lower as compared to the optimization window for the CBD (O) method (estimated to be 52 nm). This is caused by the higher doping levels obtained in Zn-polar NWs grown by the CBD method. Up to this point, the CBD (Zn) method seems to be the most limited one in comparison with the other techniques considered in this study based on the optimization window and the very low radius that is needed to obtain optimal devices.

#### 3.2.5. Electrodeposition: Analytical Evaluation of the Critical NW Radius

**Figure 7:**
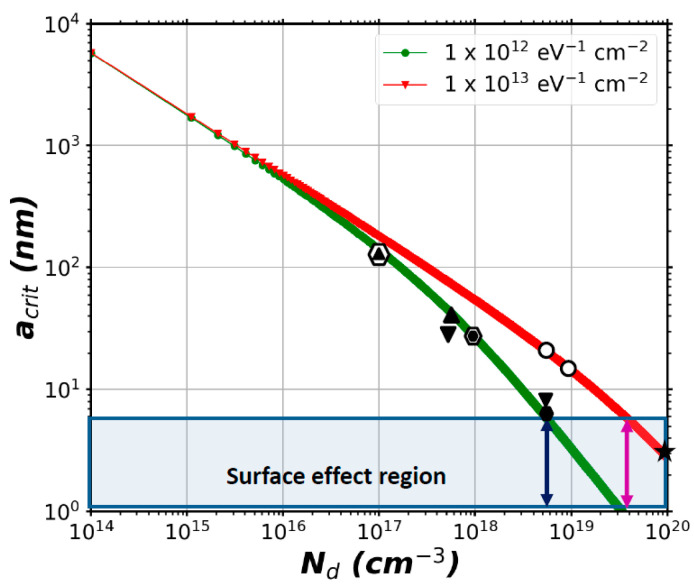
Critical radius a_crit_ as function of N_d_ for two ZnO NWs with values of N_it_ from 10^12^ (green curve) and 10^13^ eV^−1^ cm^−2^ (red curve). The black marks on the curves indicate the radius values for which full depletion was obtained as calculated using FlexPDE. The mark (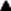) corresponds to the TE method, (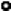) to the CBD (Zn) method, (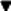) to the CBD (O) method, (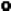) to the CVD method and (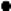) to the MOCVD method. The blue and magenta arrows indicate the limiting value of N_d_ for N_it_  = 10^12^ eV^−1^ cm^−2^ and 10^13^ eV^−1^ cm^−2^. Beyond this value of doping, the critical radius goes below 5nm and we consider that surface effects could modify piezoelectric coefficients. The mark (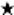) corresponds to the conditions of NWs grown by the electron-deposition method, which was not simulated with FlexPDE.

The electrodeposition method is also used to grow ZnO NWs, but it was not considered in this numerical theoretical study because of its high N_d_ (~10^20^ cm^−3^) which would lead to NWs with a wide neutral core over a wide range of NW radius. Instead, an analytical approach was considered to address the case of ZnO NWs grown by electrodeposition. To assess the agreement with the numerical simulations reported in the last sub-sections, the optimal NW radius to obtain fully depleted ZnO NWs was also calculated using an analytical model proposed in [72,73] for Si and GaAs NWs. The model considered only semiconductor equations and was developed to assess the critical radius a_crit_ (Supplementary Materials), which marks the boundary between a fully depleted NW (r < a_crit_) and a NW that is only depleted at its surface (r > a_crit_). Figure 7 shows the values calculated within the analytical model correspond very well to the values reported in the last sub-sections for the different growth methods. In the case of the CBD (O) method, the analytical value is larger compared to the values extracted from our simulations. A theoretical value of ~70 nm and ~20 nm is calculated for low and high doping concentrations. From our simulations, we extract the values of ~20 nm and ~5 nm, respectively. This difference can be explained because the analytical models do not take into account the piezoelectric effect and the orientation of the NWs. The analytical model allows us as well to estimate the possible optimal radius of NWs grown by the electro-deposition method. The values lay well below the limit of our numerical model (estimated to be 5 nm with the piezoelectric coefficients used). This confirms that our numerical model would provide inacurate piezopotential results on NWs grown by this last method.

#### 3.2.6. Effect of the Variation of NW Length on the VING Performance

**Figure 8:**
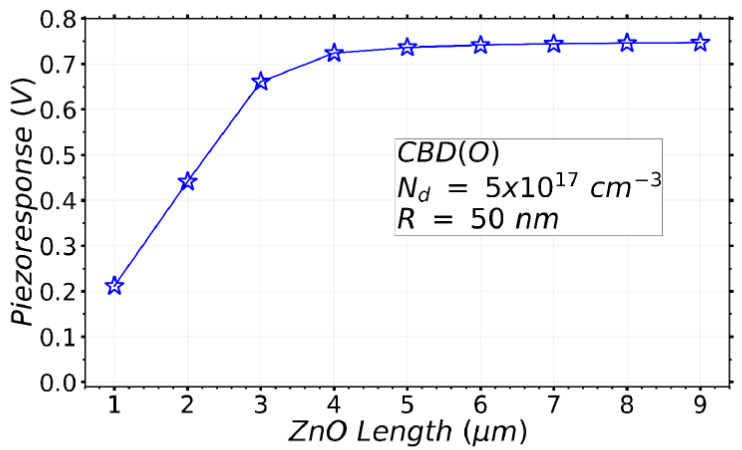
Variation of the piezoresponse of a VING device as a function of the NW length. The parameters of the NW used in the model correspond to ZnO NWs grown by the CBD (O) method.

In the simulation results from the previous sections, the piezoresponse is constant if the NW radius is smaller than a certain critical radius. This piezoresponse value is then independent upon the radius and the growth method. The value depends only on the NW length and on the ratio between the diameter of the NW and the width of the VING unit cell [54]. Here we studied the effect of the variation of the length in the particular case corresponding to the growth conditions of the CBD method forming O-polar NWs (CBD (O)). This method presents one of the largest optimization windows and is compatible with the processing of flexible devices without any additional transfer process. For this study, the NW radius was fixed at 50 nm with N_d_ = 5 × 10^17^ cm^−3^. Figure 8 shows a linear increase in the piezoresponse as the NW length increases up to 3 μm. For larger values, the piezoresponse saturates to about 0.75 V in absolute value. This means that longer NWs are not required to obtain optimal devices. This theoretical result is in accordance with the trends of experiments in energy generation [17], and output potential [18], although the output potential values are not the same, which can be due to different electrical and structural parameters. Furthermore, the inferred optimal length of O-polar NWs around 4 µm does not represent any technical challenge: it is typically achieved by the CBD method when the synthesis conditions using zinc nitrate, hexamethylenetetramine (HMTA), and chemical additives including polyethylenimine are optimized [74].

### 3.3. Summary and Discussion about the Mechanisms at Work

As a summary of these theoretical results, Table 1 shows the NW critical radius defined as the radius value below which the ZnO NW is fully depleted and optimal piezoelectric performance can be expected. The values of critical radius were calculated for every growth method studied, both for the low and high values of N_d_ allowed by each method based on reported experimental data. The range of values between the critical radii obtained for the low and high values of N_d_ defines an optimization process window. This Table shows that the different growth methods do not offer the same potential for VING device optimization when using standard conditions. The TE and CVD methods exhibit the widest optimization process window by controlling both the radius and the doping of the NWs. In particular, they allow the use of wider NWs with radius not exceeding 120 nm for the low doping level. It should be noted here that ZnO NWs grown by the TE and CVD methods present a typical radius in that range, such that the targeted critical radius is not a technological challenge. However, for the high doping level, the critical radius drops to 40 and 20 nm for the respective TE and CVD methods, respectively. In that case, the growth of ZnO NWs with this small radius is still feasible, but deserves a particular effort to be reached. On the other hand, the MOCVD method has much smaller critical radii for the low and high doping level down to 22 and 5 nm. Although the MOCVD method is a well-known technique to get high aspect ratio ZnO NWs [66], the present small radii require working in dedicated conditions such as low VI/II ratio and relatively high growth temperature [75,76]. This can be quite challenging to be reached. In contrast, ZnO NWs grown by MOCVD have significantly been developped for optoelectronic devices [77]. The CBD method has the great advantage of emphasizing the importance of considering polarity as a critical quantity for VING devices. While ZnO NWs grown by CBD with the Zn-polarity have the narrowest optimisation window and small critical radii of 18 and 12 nm for the low and high doping levels, respectively, O-polar ZnO NWs grown with the same technique exhibit one of the largest optimization window with a significant increase in the radii to obtain increased performance, half the optimal performance is reached for NWs with radii of 55 and 15 nm for the low and high doping levels. In this technique, the typical radius of ZnO NWs lies in the range of 50–100 nm when using a zinc salt as a source of zinc ions and HMTA [78]. The further decrease in the radius of ZnO NWs is more required for Zn-polar ZnO NWs than for O-polar ZnO NWs. This is typically achieved by using chemical additives such as PEI [74] and ethylenediamine [79] to inhibit the radial growth of ZnO NWs and hence to limit their radius. However, it is evident here that the addition of chemical additives is likely not sufficient for Zn-polar ZnO NWs, which points out the strong interest in developing O-polar ZnO NWs for VING devices in the capacitive configuration. It is worth noticing that these O-polar ZnO NWs also outperforms the Zn-polar ZnO NWs to get high quality Schottky contacts with Au on their top [80], which is critical in the Schottky configuration consisting in making Schottky contacts at the bottom and at the top of the VING structure [6].

In the whole of these growth methods, the reduction of the doping level to the minimum value reported here or even further may also be considered to allow larger critical radii to be used. This can tentatively be achieved by post-deposition thermal treatments including annealing under oxygen atmosphere or plasma treatment [81]. An alternative approach would consist in compensating the doping level through the introduction of acceptors [81]. The introduction of acceptors including group-I, copper and antimony in ZnO films have been investigated to a great extent [82]. However, mastering the incorporation of dopant elements in ZnO NWs is still a big issue and correlatively strongly affects their radius during the growth phase through intricate phenomena. For instance, in the CBD method, the addition of metal salts other than zinc salt offers an opportunity to dope ZnO NWs [83], but this addition is not sufficient for the incorporation of dopant elements in their center. A further precise control of the pH conditions is required [84]. The involved physicochemical processes at work also depend on the involved dopant [84,85], which makes a general approach complicated. Eventually, the engineering of surface trap density in ZnO NWs appears as an alternative way to tune the radius to a relevant range that is compatible with the different growth methods. It has been shown that the use of chemical adsorbates on the polar and non-polar planes of ZnO has a great effect on the nature and magnitude of the band bending [86,87]. The intricate nature of all these approaches represents the major difficulty to get ZnO NWs with the optimal dedicated properties. Overall, the present findings show that the different growth methods of ZnO NWs result in a different range of optimized radii and hence exhibit a different potential for VING transducers. The TE and CBD(O) methods using standard conditions are expected to be of great interest in the piezoelectric field, the latter being compatible with the processing of flexible devices without transfer processes.

## 4. Conclusions

In summary, our models predict that the performance of piezoelectric transducers based on ZnO NWs in the VING configuration evaluated under compressive forces strongly depends on the growth method. Several methods have been compared theoretically in this work: TE, CVD, MOCVD, CBD (Zn), CBD (O) and electrodeposition. Each growth method produces NWs with a different range of doping level, surface trap density, and dimensions. Taking into account these parameters in our models allowed the calculation of the optimal NW radius to obtain the best performance (i.e., maximal voltage generated for a given mechanical compression). In general, at higher doping levels, a smaller NW radius is needed to reach a full depletion of its core and to increase the performance. This effect can be compensated if the surface trap density is increased, allowing the use of wider NW radius. TE and CVD methods, with the lowest doping level, allowed the use of relatively wide NWs (radius below 120 nm) to obtain optimal results. MOCVD method required relatively thinner NWs compared to the other methods to improve the performance of the transducers (radius below 22 nm at low doping level). The CBD(O) method is the only other method allowing relatively large radius (below 55 nm) at low doping level thanks to the higher surface trap density. The CBD (Zn) method requires a thinner radius for optimal devices compared to the other methods studied here (below 18 nm and 12 nm at low and high doping level, respectively). This method also presents the smallest window of optimisation in terms of optimal radius. Numerical calculations have been compared with an analytical approach to obtain the optimal radius. We found a very good correspondence between the two calculation approaches. The analytical approach was used as well in the highest doping level range corresponding to electrodeposition. The optimal NW radius for this method could be below 5 nm, suggesting a more complex model where piezoelectric properties are taken into account as a function of surface parameters. Our numerical model also predicts that an increase in the NW length increases device performance until a saturation in the output potential is reached. This means that a certain minimum length is needed to optimize the device. As an example, in the case of CBD (O) grown ZnO NWs with a radius of 50 nm and low doping level, increasing the length beyond 4 µm does not improve anymore the performance. Doping level and surface and interface traps will also affect piezotronic and piezo-phototronic devices and it would be very important to take into account the NW growth method on those applications as well. Finally, the control of surface and interface traps densities by surface engineering is key to optimize the performance of piezoelectric transducers based on piezoelectric semiconducting NWs.

## Figures and Tables

**Table 1:** Summary of NW radius values to achieve full depletion or optimal performance for the different growth methods (NW critical radius). In the case of the CBD (O) technique, the values correspond to the radius to obtain half the optimal performance.

Growth Method	ZnO NW Radius for Full Depletion	
	$\mathrm{Min}.\;N_{d}\;(\mathrm{nm})$	$\mathrm{Max}.\;N_{d}\;(\mathrm{nm})$
**TE**	<120	<40
**CVD**	<120	<20
**MOCVD**	<22	<5
**CBD (O)**	<55	<15
**CBD (Zn)**	<18	<12
**Electrodeposition**	Not simulated (estimated < 4)	Not simulated (estimated < 4)

## Data Availability

The data are available upon request from the corresponding authors.

## Supplementary Materials

The following are available online at https://www.mdpi.com/article/10.3390/nano11040941/s1, Figure S1: Energy Band diagram along half the cross-section of a n-type ZnO NW.
