# Supplementary material for: Dimensional Roadmap for Maximizing the Piezoelectrical Response of ZnO Nanowire-Based Transducers: Impact of Growth Method
Source: Nanomaterials (Basel). 2021 Apr 7;11(4):941. doi: 10.3390/nano11040941 (PMC8067815; doi:10.3390/nano11040941)
Supplement: Supplementary file 1 [file nanomaterials-11-00941-s001.pdf]

Supplementary Materials

Dimensional Roadmap for Maximizing the Piezoelectrical Response of ZnO Nanowire-Based Transducers: Impact of Growth Method

Andrés Jenaro Lopez Garcia [1], Mireille Mouis [1], Vincent Consonni [2] and Gustavo Ardila [2,*]

[1] Univ. Grenoble Alpes, CNRS, Grenoble INP, IMEP-LaHC, F-38000 Grenoble, France; andres-jenaro.lopez-garcia@grenoble-inp.fr (A.J.L.G.); mouis@minatec.grenoble-inp.fr (M.M.)
[2] Univ. Grenoble Alpes, CNRS, Grenoble INP, LMGP, F-38000 Grenoble, France; vincent.consonni@grenoble-inp.fr

* Correspondence: ardilarg@minatec.grenoble-inp.fr; Tel.: +33.4.56.52.95.32

The calculation of the critical radius follows the same line as in [1].

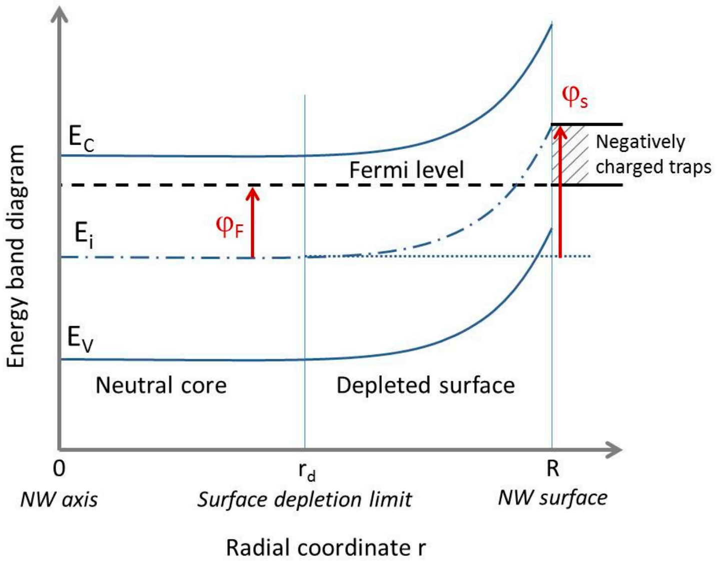

Figure S1. Energy Band diagram along half the cross-section of a n-type ZnO NW (Adaptated picture from supporting information [2]).

The charge density surface Q_s is always applicable on a cross section of a cylinder with a surface of $2\pi a$, where a is the radius. Consequently, Q_s can be determined as

$$Q_s = -q \cdot N_{it} \cdot (\varphi_s - \varphi_F), \quad \text{(S1)}$$

Where q is the electron charge, N_{it} is surface traps density, φ_s is the surface potential and φ_F is the difference between the Fermi level and intrinsic level as shown the in Figure S1. Assuming the full depletion approximation (i.e., $n, p \ll N_d$), the electric charge density ρ can be given by

$$\rho = \begin{cases} 0 & 0 \leq r < r_d \\ qN_D & r_d \leq r \leq a \end{cases} \quad \text{(S2)}$$

Solving the Poisson's equation in cylindrical coordinates and using the boundary condition according to the parameters given on Figure S1, we can write $\varphi_s(a_{crit})$ at the limit when r_d tends to 0 as

$$\varphi_s = \frac{qN_d}{4\varepsilon} \cdot {a_{crit}}^2 \quad \text{(S3)}$$

Applying the charge neutrality condition at the surface between Q_s and the definition of depletion charge Q_{dep} given by

$$Q_{dep} = qN_d \cdot \pi {a_{crit}}^2 \quad \text{(S4)}$$

Finally, we can obtain the critical radius a_c as function of N_{it} and N_d for a fully depleted NW and it is given by

$$a_{crit} = \frac{\varepsilon}{qN_{it}} \left[-1 + \sqrt{1 + \frac{4kT}{\varepsilon} \frac{{N_{it}}^2}{N_d} \cdot \ln \frac{N_d}{n_i}} \right] \quad \text{(S5)}$$

REFERENCES:

1. Schmidt, V.; Senz, S.; Gösele, U. Influence of the Si/SiO2 interface on the charge carrier density of Si nanowires. *Appl. Phys. A Mater. Sci. Process.* **2007**, 86, 187–191.

2. Tao, R.; Mouis, M.; Ardila, G. Unveiling the Influence of Surface Fermi Level Pinning on the Piezoelectric Response of Semiconducting Nanowires. *Adv. Electron. Mater.* **2018**, 4, 1–9.
